# Type III Nrg1 Back Signaling Enhances Functional TRPV1 along Sensory Axons Contributing to Basal and Inflammatory Thermal Pain Sensation

**DOI:** 10.1371/journal.pone.0025108

**Published:** 2011-09-20

**Authors:** Sarah E. Canetta, Edlira Luca, Elyse Pertot, Lorna W. Role, David A. Talmage

**Affiliations:** 1 Department of Neurobiology and Behavior, Columbia University, New York, New York, United States of America; 2 Integrated Department, Columbia University, New York, New York, United States of America; 3 Department of Neurobiology and Behavior, State University of New York at Stony Brook, Stony Brook, New York, United States of America; 4 Department of Biological Science, State University of New York at Stony Brook, Stony Brook, New York, United States of America; 5 Center for Nervous System Disorders, State University of New York at Stony Brook, Stony Brook, New York, United States of America; 6 Department of Pharmacological Science, State University of New York at Stony Brook, Stony Brook, New York, United States of America; Medical College of Georgia, United States of America

## Abstract

Type III Nrg1, a member of the Nrg1 family of signaling proteins, is expressed in sensory neurons, where it can signal in a bi-directional manner via interactions with the ErbB family of receptor tyrosine kinases (ErbB RTKs) [1]. Type III Nrg1 signaling as a receptor (Type III Nrg1 back signaling) can acutely activate phosphatidylinositol-3-kinase (PtdIns3K) signaling, as well as regulate levels of α7* nicotinic acetylcholine receptors, along sensory axons [2]. Transient receptor potential vanilloid 1 (TRPV1) is a cation-permeable ion channel found in primary sensory neurons that is necessary for the detection of thermal pain and for the development of thermal hypersensitivity to pain under inflammatory conditions [3]. Cell surface expression of TRPV1 can be enhanced by activation of PtdIns3K [4], [5], [6], making it a potential target for regulation by Type III Nrg1. We now show that Type III Nrg1 signaling in sensory neurons affects functional *axonal* TRPV1 in a PtdIns3K-dependent manner. Furthermore, mice heterozygous for Type III Nrg1 have specific deficits in their ability to respond to noxious thermal stimuli and to develop capsaicin-induced thermal hypersensitivity to pain. Cumulatively, these results implicate Type III Nrg1 as a novel regulator of TRPV1 and a molecular mediator of nociceptive function.

## Introduction

Type III Nrg1, a member of the Nrg1 family of signaling proteins, is expressed in sensory neurons where it has been shown to be important for receptor localization as well as nociceptive sensory neuron survival [2], [7]. The Nrg1 gene has also been identified as a candidate schizophrenia susceptibility gene [8], [9], [10], and levels of the Type III isoform were reported to be decreased in post-mortem tissue of some schizophrenics [11]. Schizophrenia patients are impaired in their ability to perceive painful stimuli, particularly under inflammatory conditions [12], [13], [14], [15], [16], [17], [18]. We therefore investigated whether reducing levels of Type III Nrg1 alters response to nociceptive stimuli under normal and inflammatory conditions.

Transient receptor potential vanilloid 1 (TRPV1) is a cation-permeable ion channel found in primary sensory neurons that contributes to the detection of thermal pain and is necessary for the development of thermal hyperalgesia under inflammatory conditions [3], [19]. Membrane surface expression of TRPV1 can be enhanced by activation of phosphatidylinositol-3-kinase (PtdIns3K) [4], [5], [6] and increased levels of functional surface TRPV1 along sensory neuron axons contribute to behavioral sensitization to pain [20].

Type III Nrg1 can signal in a bi-directional manner after interactions with the ErbB family of receptor tyrosine kinases (ErbB RTKs). Type III Nrg1 can serve as a ligand to activate ErbB RTKs and initiate signaling in ErbB-expressing cells [21]. Additionally, Type III Nrg1 can signal as a receptor when stimulated by interaction with ErbB RTKs or depolarization [1], [22]; Type III Nrg1 signaling as a receptor (Type III Nrg1 back signaling) can have both transcriptional and non-transcriptional effects [1], [2], [22], [23]. In particular, Type III Nrg1 back signaling can acutely activate PtdIns3K along sensory neuron axons [2] making TRPV1 a potential molecular link between Type III Nrg1, schizophrenia and alterations in pain sensation.

We now show that mice heterozygous for Type III Nrg1 [7] have specific deficits in their ability to respond to noxious thermal stimuli, and in their ability to develop capsaicin-induced thermal hypersensitivity to pain. These particular behavioral deficits are reminiscent of mice that lack the receptor TRPV1 [3], [19]. A reduction in levels of Type III Nrg1 is associated with a reduction in functional TRPV1 along sensory neuron axons. Furthermore, acute stimulation of Type III Nrg1 back signaling in WT sensory neuron axons leads to an increase in functional TRPV1 in a phosphatidylinositol-3-kinase (PtdIns3K)-dependent manner. In addition to shedding insight into the function of Type III Nrg1 in sensory neurons, these findings provide a potential biological mechanism for some of the abnormalities in pain sensation seen in patients with schizophrenia.

## Materials and Methods

### Animals

All experiments were conducted in accordance with National Institutes of Health *Guide for the Care and Use of Laboratory Animals* and studies were approved by Institutional Animal Care and Use Committees at Columbia University (#3132) and Stony Brook University (#1618 and #1792). Mice heterozygous for an isoform specific disruption of Type III Nrg1 (*Nrg1^tm1Lwr^*) and their wild-type littermates were generated and genotyped as previously described [7]. Animal lines were back-crossed onto a C57/Bl6 background. WT and Type III Nrg1 heterozygous (Type III Nrg1^+/−^) littermates were generated from heterozygote crosses. TRPV1^−/−^ mice were obtained from Jackson Labs. As TRPV1^−/−^ mice are viable and fertile, TRPV1^−/−^ crosses were used to generate the TRPV1^−/−^ mice used in experiments. Only male mice were used for experiments unless specifically stated. Animals were housed 2–4 per cage, maintained on a 12 h light/dark cycle and provided with food and water *ad libitum*.

### Cell culture

Primary DRG cultures were prepared from P21 WT or Type III Nrg1^+/−^ male mice. Lumbar DRGs were dissected into L-15 media (Sigma) and incubated in a 0.1% collagenase (Type IV, Worthington) solution for 1 hour at 37°C/5% CO_2_. Cells were rinsed once in L-15 before being dispersed in MEM (Gibco) supplemented with 10% Fetal Bovine Serum (Gemini Bio-Products), 50 u/ml each of penicillin and streptomycin (Gibco), 2 mM L-glutamine (Gibco) and 50 ng/ml NGF (Harlan Bioscience) using a series of fire-polished pipettes. For immunostaining and calcium imaging, cells were plated onto 12-mm nitric-acid washed, poly-D-lysine (1 mg/ml, Chemicon) and laminin (100 µg/ml, Sigma) coated glass coverslips (Warner Instruments) at a density of ∼4,000 cells/well. Cells were left at 37°C/5% CO_2_ for 36–48 hours before use. For immunoblots, cells were plated onto poly-D-lysine and laminin coated cell culture plates (6-well plate: 20,000 cells/well; 12-well plate: 10,000 cells/well) and left at 37°C/5% CO_2_ for 48 hours before use.

### Reagents

Where indicated, the following reagents were applied to cell cultures: Capsaicin (Sigma; prepared as a 100 mM stock solution in 10% ethanol), capsazapine (Tocris Bioscience; prepared as a 10 mM stock in 70% EtOH), 300 ng/ml soluble ErbB4-ECD (sErbB4-ECD) (Bao et al. 2003), 2.5S nerve growth factor (NGF; BT-5025 Harlan Bioproducts), 20 nM wortmannin (WM; Calbiochem), 50 µM LY294002 (Cell Signaling Technology) and 1 µM PD158780 (Calbiochem).

### Behavior

Male WT and Type III Nrg1^+/−^ littermates, 5–10 months of age, were used for behavioral tests. The experimenter was blind to the genotype of the mice during testing.

### Hargreaves Paw Withdrawal Assay

Heat pain threshold of the plantar hindpaw was assessed using the Hargreaves method [24]. Animals were placed in pairs in plastic enclosures on an elevated glass plate pre-warmed to 30°C and allowed to acclimate for 1.5 hours. A focused beam of light with the percent intensity set to 15 (IITC Life Science) was used to heat the plantar surface of each hindpaw, and the time for the animals to withdraw their hindpaws from the light source was recorded to the hundredth of a second as the reaction latency. For baseline reaction latency measurements each paw was tested 4 separate times on 2 separate days and the values for each paw were averaged together by animal. Averages for reaction latency were compared by genotype.

### Cold Plate Test

Noxious cold sensitivity of the plantar surface of the hindpaw was assessed using a cold plate analgesia meter (IITC Life Science). To measure pain, mice were placed on the metal surface pre-set to 0°C, and the latency to generate a nocifensive response (lifting or flicking the hindpaws or jumping), as well as the total number of nocifensive responses generated within the five-minute interval in which the animal remained on the plate, were recorded. Averages for each variable were compared by genotype.

### von Frey Mechanosensitivity Test

Mechanical withdrawal thresholds of the plantar hindpaw were assessed using the von Frey method. Animals were placed on a plastic grid in plastic enclosures and allowed to acclimate for 20 minutes. Filaments of increasing durability (IITC Life Science) were used to apply escalating levels of force to the plantar hindpaw. Each filament was applied 5 times to the hindpaw and the number of times the animal withdrew its paw from that stimulus intensity was recorded and expressed as a percent response. Average percent response at each stimulus intensity was compared by genotype.

### Modified Hargreaves Paw Withdrawal Assay with Capsaicin Application

A modified version of the Hargreaves method was used to assess the development of inflammatory thermal hypersensitivity in the plantar hindpaw following application of capsaicin. The Hargreaves Assay was used as described above, with the following modifications. The glass plate was left at room temperature (25°C) and the percent intensity of the light source was set to 10 (IITC Life Science). Baseline measurements were made testing each paw 4 times and the paw withdrawal latencies were averaged by paw. The following day, animals were lightly anesthetized with 2% isofluorane, and 20 µl of 0.075% capsaicin cream (Zostrix) was applied to the right hindpaw and lightly massaged into the skin. The animals were allowed to recover on the elevated glass plate in plastic enclosures for 20 minutes before reaction latency was again assessed for each paw. Absolute reaction latencies, as well as percent changes from baseline, were compared by genotype.

### Histology

For immunofluorescent or immunohistochemical staining of DRG tissue, adult male mice were deeply anesthetized with a mixture of ketamine-xylazine (90 mg/kg and 10 mg/kg, respectively) and transcardially perfused with 0.1 M PBS for 2 minutes followed by 4% paraformaldehyde (PFA) in PBS for 2 minutes. Following perfusion, L4 and L5 DRG were dissected out bilaterally, post-fixed in 4% PFA overnight, embedded in paraffin and cut in 7 µm thick sections on a microtome. Serial sections from litter matched adult male WT and Type III Nrg1^+/−^ mice were collected on Superfrost plus microscope slides such that each slide contained representative sections taken approximately every 150 µm throughout the entire DRG. To visualize DRG cell profiles, paraffin-embedded tissue was subjected to antigen retrieval, blocked in 10% normal donkey serum (NDS) in PBS with 0.1% TritonX-100 (PBS-T) for 30 minutes at room temperature, and left in primary antibody diluted in PBS-T overnight at 4°C. Primary antibodies used for cell counting include those against TrkA (rabbit, 1∶2000, gift of L.F. Reichardt), peripherin (rabbit, 1∶1000, ab1530 Millipore and mouse, 1∶1000, ab4573 abcam), and TRPV1 (guinea pig, 1∶1000, GP14100 Neuromics), as well as the marker lectin IB4-FITC (1∶200 in PBS containing Ca^2+^, L9381 Sigma). Type III Nrg1 was detected using a Type III-specific antibody generated in our lab (host = chicken) that recognizes an extracellular epitope of the protein (peptide sequence: ARTPEVRTPKSGTQPQTTET, corresponding to residues 195–214 of the Type III Nrg1 protein, KHL conjugated, Pocono Rabbit Farm & Laboratory Inc). Specific staining of motor neurons in the ventral horn of the spinal cord was used as a positive control (data not shown). This staining disappeared when the antibody was pre-incubated with the peptide against which it was generated, confirming the specificity of the staining in adult tissue (data not shown). Serum samples taken from the animal before it was immunized were used as a negative control and did not stain the spinal cord, dorsal root ganglia or hindpaw skin (data not shown). The following day secondary antibodies conjugated to Biotin (1∶500, Jackson ImmunoResearch), Alexa488 (1∶500, Molecular Probes), Alexa594 (1∶500, Molecular Probes), Alexa546 (1∶500, Molecular Probes) or AMCA (1∶50, Jackson ImmunoResearch) (diluted in 10% NDS in PBS-T) were applied for 1 hour at room temperature. For immunofluorescence, sections were subsequently coverslipped using Vectashield (Vector Laboratories Inc, generally containing DAPI) and sealed with nail polish. For immunohistochemistry, sections were incubated with Avidin-Biotin Complex (ABC, Vector Laboratories Inc) and developed using a Diaminobenzidine (DAB) reaction (Vector Laboratories Inc), before being dehydrated and cleared with ethanol and xylene washes and mounted using Permount (Fisher Scientific). Co-localization of Type III Nrg1 with various sensory markers was assessed by acquiring confocal images of DRG sections using the Olympus Spinning Disk Confocal microscope (DSU; Olympus) equipped with a 40× oil Plan ApoN objective (1.42 NA), electron-multiplying charge-coupled device camera (Hamamatsu), and Slidebook software (Version 5; Olympus).

To count DRG neuronal profiles immunoreactive for a particular sensory marker, epifluorescent images were acquired using an Axio Imager microscope (Carl Zeiss, Inc.) equipped with a 20× Plan-Apochromat objective (0.8 NA), a charge-coupled device camera (Hamamatsu) and Metamorph software (MDS Analytical Technologies). A constant exposure time was used for each sensory marker. Images were analyzed using Metamorph by creating constant intensity thresholds above which cells were counted as positively stained. Two independent observers tallied the number of neuronal profiles positively stained for both the specific and pan-sensory marker in each tissue section. The results from both observers were averaged and used to generate percentages of sensory neurons staining positively for individual sensory markers. For each marker, counts from at least five tissue sections per animal were averaged. 3 animals per genotype were analyzed and genotype averages were compared.

For hindpaw staining, deeply anesthetized male mice were transcardially perfused with PBS for 4 minutes, plantar skin from the hindpaw was dissected out and post-fixed in 4% PFA for 18 hours before being cryoprotected in 30% sucrose. Tissue was embedded in OCT mounting media and stored at −80°C until sectioning. 20 µm sections were sectioned serially on a cryostat and stored at −20°C until needed. For TRPV1 hindpaw staining, mice were given a plantar injection of 1 µg of NGF (in 10 µl saline) 48 hours prior to transcardial perfusion. Further tissue preparation was as described above, except for the use of 40 µm cryostat sections. For immunofluorescent staining, sections were rehydrated in PBS-T and blocked in 10% NDS in PBS-T for 1.5 hours at room temperature before primary antibodies were applied overnight at 4°C. Primary antibodies used to stain the hindpaw include those against CGRP (rabbit, 1∶1000, T-4032 Bachem), TRPV1 (guinea pig, 1∶1000, GP14100 Neuromics) and Type III Nrg1 (chicken, 1∶2000). The following day, secondary antibodies conjugated to Alexa488 or Alexa546 (1∶500, Molecular Probes) (diluted in 10% NDS in PBS-T) were applied and sections were coverslipped with Vectashield (containing DAPI) and sealed with nail polish.

For immunofluorescent staining of primary DRG cultures, cells grown for 36–48 hours were rinsed twice in PBS, fixed in 4% PFA, permeabilized in PBS-T for 10 minutes and blocked in 10% NDS in PBS for 1 hour at room temperature before incubation with primary antibody. To assess activation of PtdIns3K along WT and Type III Nrg1^+/−^ axons, cultures were serum-starved for 4 hours, and then treated with either sErbB4-ECD (300 ng/ml) or NGF (100 ng/ml) for 15 minutes at 37°C before being stained with antibodies against pAKT (Ser473, rabbit, 1∶200, 4060 Cell Signaling Technology), Type III Nrg1 (chicken, 1∶2∶000) and pan-axonal proteins (mouse, 1∶500, SMI-312R Covance) overnight at 4°C followed by secondary detection with Alexa488-, Alexa594- or AMCA-conjugated secondary antibodies (diluted in 10% NDS in PBS). Fields of view containing axons selected based on their expression of Type III Nrg1 and pan-axonal protein were imaged using epifluorescence, and photographed with a constant exposure time using an Axio Imager microscope (Carl Zeiss, Inc.) equipped with a 63× oil Plan-Apochromat objective (1.4 NA), a charge-coupled device camera (Hamamatsu), and Metamorph software (MDS Analytical Technologies). At least 5 fields of view were acquired from each of the three animals per genotype. Briefly, axons were outlined in Metamorph using the pan-axonal marker as a guide, the outlined regions were transferred to the pAKT image, and the average fluorescence intensity (AFI) of the pAKT staining along these regions was recorded. Intensity of pAKT staining was averaged by animal within a given treatment and average pAKT intensity was compared by genotype and treatment.

To quantify levels of Type III Nrg1 along P21 WT and Type III Nrg1^+/−^ axons, cultures were treated with antibodies that recognized either an extra-cellular epitope of Type III Nrg1 (chicken, 1∶2000, 2 hours at 37°C) or the C-terminal intracellular domain (Nrg1-ICD, rabbit, 1∶200, sc348 Santa Cruz, overnight at 4°C). Cultures were also stained for peripherin (rabbit or mouse, 1∶1000), before secondary detection with Alexa488- or Alexa594-conjugated secondary antibodies (diluted in 10% NDS in PBS). Fields of view containing axons (identified based on expression of peripherin) were imaged using epifluorescence using a 63× oil Plan-Apochromat objective (1.4 NA) and photographed with a constant exposure time. At least 5 images were acquired from each of the three animals per genotype. Images were processed with MetaMorph software. Axons were outlined using peripherin staining as a guide, and the number of Type III Nrg1+ and Nrg1-ICD+ punctae along these peripherin+ axons with intensity greater than a defined threshold value were quantified and expressed as the number of punctae per 100 µm of axon length. These values were averaged within a given image and compared by genotype.

### Calcium Imaging

At 3 days in vitro, primary DRG cultures were loaded with 5 µM Fluo-4 Ca^2+^ binding dye (Invitrogen) in 1× HEPES Buffered Saline (HBS) with 2 µM Pluronic-F127 (Invitrogen) for 15 minutes at 37°C/5% CO_2_. Cells were rinsed 2 times with HBS and left to rest at 37°C/5% CO_2_ for 30 minutes before use. During imaging, cells were perfused with HBS containing 2 µM tetrodotoxin (TTX, Tocris), 10 µM bicuculline (Tocris), 50 µM D-(−)-2amino-5-phosphono-valeric acid (D-APV, Tocris) and 20 µM 6-Cyano-7-nitroquinoxaline-2,3-dione (CNQX, Tocris) at a rate of 0.5 ml/minute. Images were captured every 3 seconds using a spinning disc confocal microscope (DSU; Olympus) equipped with a 40× oil Plan ApoN objective (1.4 NA), electron-multiplying charge-coupled device camera (Hamamatsu), and Slidebook software (Version 5; Olympus). After acquiring images for 1 min, 1 µM of capsaicin was focally applied by pressure ejection (Picospritzer II, General Valve Corporation) for 20 seconds. Depending on the experiment, capsaicin was applied between 1 and 4 additional times. At the end of each experiment, the cultures were depolarized by application of 56 mM KCl for 20 seconds. Regions (2.5 by 2.5 µm) were drawn along axons that responded to both capsaicin and KCl and the percent change in fluorescence intensity from baseline ([(F−F_0_)/F_0_]*100) was calculated within these regions using MetaMorph software. All regions were averaged by axon; all axons were averaged by animal and animal averages were compared by genotype and treatment. For analysis of somal responses, circular regions were drawn just inside the boundaries of the cell body and [(F−F_0_)/F_0_]*100 was calculated within these regions as described above.

### Immunoblotting

#### For TRPV1, DRG

P21 WT and Type III Nrg1^+/−^ DRG cultures were grown in a 6-well plate, rinsed 2 times on ice with PBS, lysed into a modified RIPA buffer (95 mM NaCl, 25 mM Tris pH 7.4, 10 mM EDTA, 1 mM EGTA, 10 µM ammonium molybdate, 1 µM sodium orthovanadate, 1% SDS and protease inhibitors) and homogenized using a pipette tip. Lysate was left on ice for 10 minutes followed by centrifugation at 10,000 rpm for 10 minutes at 4°C. Lysate was mixed with Laemmli buffer, heated at 37°C, separated on a 7.5% SDS-PAGE gel and transferred to a nitrocellulose membrane (Whatman). The membrane was then blocked for 1 hour in 5% milk (Carnation)/Tris Buffered Saline (TBS) and antibodies directed against TRPV1 (rabbit, 1∶1000, RA14113 Neuromics) and GAPDH (mouse, 1∶5000, CB1001 Calbiochem) were applied in 5% milk/Tris Buffered Saline with 0.1% Tween-20 (TBS-T) overnight at 4°C. After three 10-minute rinses in TBS-T at room temperature, secondary antibodies conjugated to Alexa680 or Alexa800 (Molecular Probes) or IRDye-700 or 800 (Rockland) were applied at a concentration of 1∶5000 diluted in 5% milk/TBS-T for 1 hour at room temperature. Membranes were scanned with an Odyssey Infrared Imaging System (Li-Cor Biosciences) and band intensities were analyzed using Odyssey Image Analysis software. The intensity of the TRPV1 band was normalized to that of GAPDH to control for variable protein loading. TRPV1-GAPDH ratios were compared by genotype.

#### For pAKT and pERK, DRG

P21 WT and Type III Nrg1^+/−^ cultures grown in a 12-well culture plate for 36–48 hours were serum starved for 4 hours before being treated with sErbB4-ECD (300 ng/ml for 15 minutes) or NGF (100 ng/ml for 5 minutes) at 37°C, rinsed 2 times on ice with TBS and homogenized with a pipette tip in lysis buffer (1% Ipegal, 30 mM HEPES, 100 mM NaCl, 25 mM NaF, 10 mM EDTA, 4 mM EGTA, 15 mM sodium pyrophosphate, 10% glycerol, protease and phosphatase inhibitors, 1 mM PMSF and 5 mM sodium orthovanadate). The lysate was left on ice for 10 minutes followed by centrifugation at 10,000 rpm for 10 minutes at 4°C. Lysate was mixed with Laemmli buffer, heated to 90°C for 10 minutes, separated on a 10% SDS-PAGE gel and transferred to a nitrocellulose membrane. The membrane was then blocked for 1 hour in 5% milk/TBS and antibodies directed against pAKT (Ser473, rabbit, 1∶1000, 9271 Cell Signaling Technology), pERK (Thr202/Tyr204, mouse, 9106 Cell Signaling Technology) and GAPDH were applied in 5% milk/TBS-T overnight for 2 nights at 4°C. Secondary antibodies conjugated to Alexa680 or IRDye-700 were applied at a concentration of 1∶5000 in 5% milk/TBS-T for 1 hour at room temperature and bands were detected by scanning with the Odyssey Infrared Imaging System. Subsequently, membranes were re-blocked in 5% milk/TBS-T and incubated in antibodies directed against AKT (rabbit, 1∶1000, 9272 Cell Signaling Technology) and ERK (rabbit, 1∶1000, 9102 Cell Signaling Technology) diluted in 5% milk/TBS-T overnight at 4°C followed by detection with secondary antibodies conjugated to Alexa800 or IRDye-800 for one hour at room temperature. Membranes were rescanned using the Odyssey system and band intensities were analyzed using Odyssey Image Analysis software. Ratios of pAKT-AKT and pERK-ERK were determined for each sample and compared by treatment and/or genotype.

#### For TRPV1, TrkA and Ret, Hindpaw

P21 WT and Type III Nrg1^+/−^ hindpaw galabrous skin samples were lysed into a modified RIPA buffer (95 mM NaCl, 25 mM Tris pH 7.4, 10 mM EDTA, 1 mM EGTA, 10 µM ammonium molybdate, 1 µM sodium orthovanadate, 1% SDS and protease inhibitors) and homogenized using a dounce homogenizer. Lysate was left on ice for 10 minutes followed by centrifugation at 10,000 rpm for 10 minutes at 4°C. Lysate was mixed with Laemmli buffer, heated at 95°C, separated on a 7.5% SDS-PAGE gel and transferred to a nitrocellulose membrane (Whatman). The membrane was then blocked for 1 hour in 5% milk (Carnation)/Tris Buffered Saline (TBS) and antibodies directed against TRPV1 (rabbit, 1∶500, RA14113 Neuromics) and GAPDH (mouse, 1∶5000, CB1001 Calbiochem) or TrkA (rabbit, 1∶500, gift of L.F. Reichardt), Ret (goat, 1∶500, GT15002) and GAPDH (mouse, 1∶5000, CB1001 Calbiochem) were applied in 5% milk/Tris Buffered Saline with 0.1% Tween-20 (TBS-T) overnight at 4°C. After three 10-minute rinses in TBS-T at room temperature, secondary antibodies conjugated to Alexa680 or Alexa800 (Molecular Probes) or IRDye-700 or 800 (Rockland) were applied at a concentration of 1∶5000 diluted in 5% milk/TBS-T for 1 hour at room temperature. Membranes were scanned with an Odyssey Infrared Imaging System (Li-Cor Biosciences) and band intensities were analyzed using Odyssey Image Analysis software. The intensity of the TRPV1, TrkA and Ret bands were normalized to that of GAPDH to control for variable protein loading. TRPV1, TrkA or Ret-GAPDH ratios were compared by genotype.

### RT-PCR Analysis of Type III Nrg1 Splice Variants

Relative levels of Type III Nrg1 transcripts containing the TMc exon versus those containing the β3 exon were measured by semi-quantitative RT-PCR. Total RNA from thoracic, lumbar and sacral level DRG from P21 male mice was purified using Trizol and 2 µg was converted to cDNA using Superscript III (Invitrogen). 0.5 µg of each cDNA was amplified using either a CRD 5′ and TMc 3′ primer pair, a CRD 5′ and β3 3′ primer pair, or all three primers together (primer concentrations in each instance were 200 ng). The resulting products were resolved on an agarose gel and relative ethidium fluorescence was quantified. The predicted product sizes for the CRD – TMc product was 359 bp (assuming the β1 splice form was amplified) and 309 bp for the CRD-β3 Primer sequences used were: CRD5′CAGGAACTCAGCCACAAACA; TMc 3′: TAGGCCACCACACACATGAT, β3 3′: ACAAGAAAGCAGCACCGACT.

### Statistical Significance

Normally distributed data was evaluated using a Student's t-test or an ANOVA with a Holm-Sidak or Fischer's PLSD post-hoc test for multiple comparisons. Non-normally distributed data was compared using a Mann-Whitney Rank Sum test or an ANOVA on Ranks with a Dunn's post-hoc test for multiple comparisons. Statistical significance was set at p<0.05.

## Results

### Type III Nrg1 is found in adult nociceptive sensory neuron soma and peripheral nerve terminals

**Figure 1:**
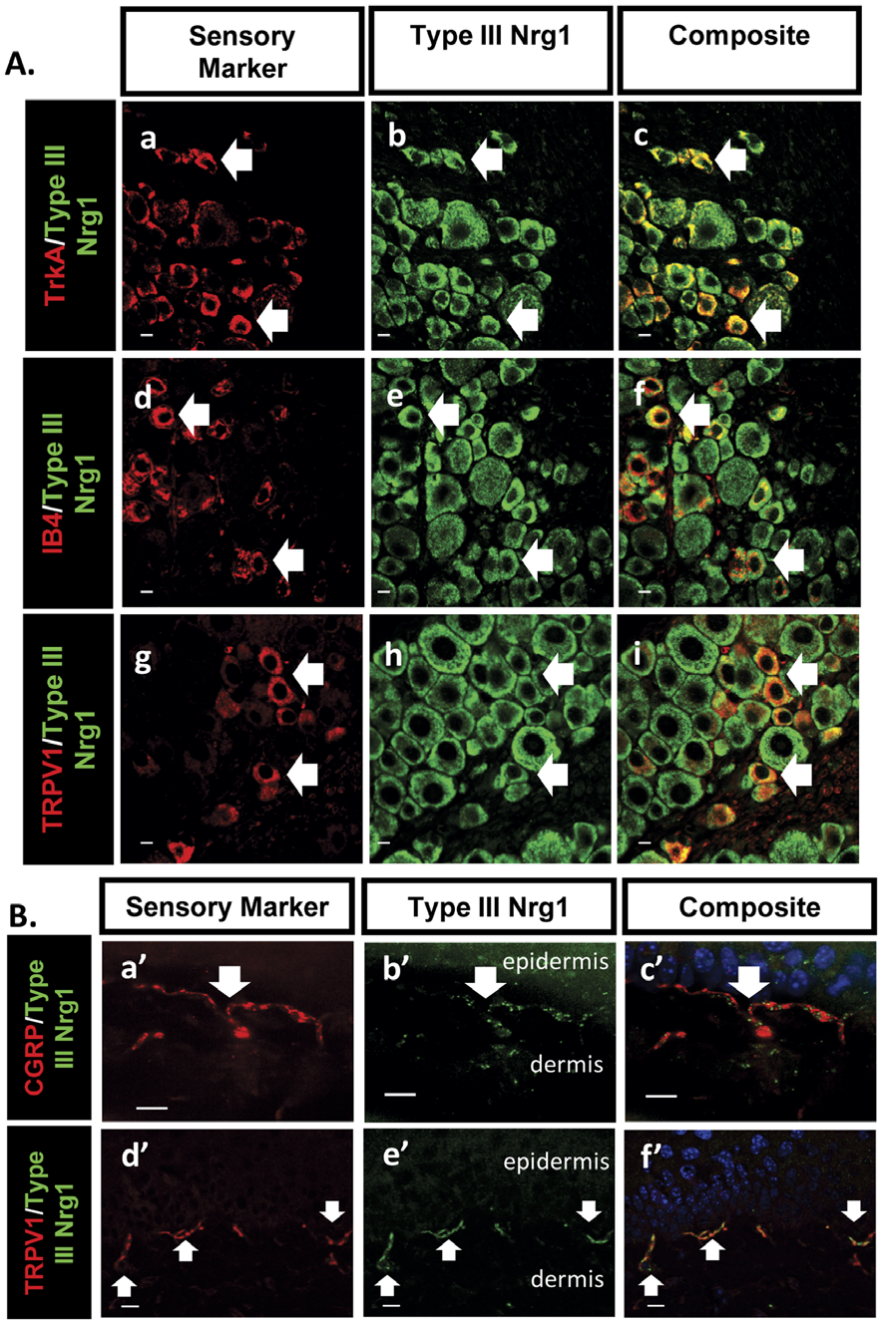
Type III Nrg1 expression in adult nociceptive sensory neuron soma and peripheral nerve terminals in vivo. (A) Type III Nrg1 (b,e,h) is found in nociceptive TrkA+ (a–c) and IB4+ (d–f), as well as TRPV1+ (g–i) sensory neuron soma in lumbar dorsal root ganglia. (B) Type III Nrg1 (b′,e′) is found in nociceptive peripheral nerve terminals innervating the plantar hindpaw skin as marked with an antibody against CGRP (a′) as well as TRPV1 (d′). White arrows indicate examples of soma or nerve terminals that are expressing both Type III Nrg1 and the particular sensory marker. All scale bars equal 10 µm.

In order to determine the pattern of Type III Nrg1 expression in nociceptive peripheral sensory neurons of the adult mouse, we used double immunofluorescent labeling of transverse sections through mouse lumbar DRG (Figure 1A) and plantar hindpaw skin (Figure 1B). In L4/L5 DRG Type III Nrg1 protein is expressed in both major classes of nociceptive sensory neurons, the peptidergic nociceptive sensory neurons (TrkA; Figure 1A(a–c)) and the non-peptidergic nociceptive sensory neurons (IB4; Figure 1A(d–f)). Type III Nrg1 is also found in the population of nociceptive sensory neurons that expresses the thermoreceptor, TRPV1 (Figure 1A(g–i)). Additionally, we found punctate Type III Nrg1 expression along nociceptive peripheral nerve terminals innervating the plantar skin of the hindpaw as evidenced by Type III Nrg1 co-staining with both the peptidergic marker, CGRP (Figure 1B(a′–c′), white arrow) as well as TRPV1 (Figure 1B(d′–e′), white arrows).

### Heat pain and thermal hypersensitivity to pain are reduced in the Type III Nrg1^+/−^ mouse

**Figure 2:**
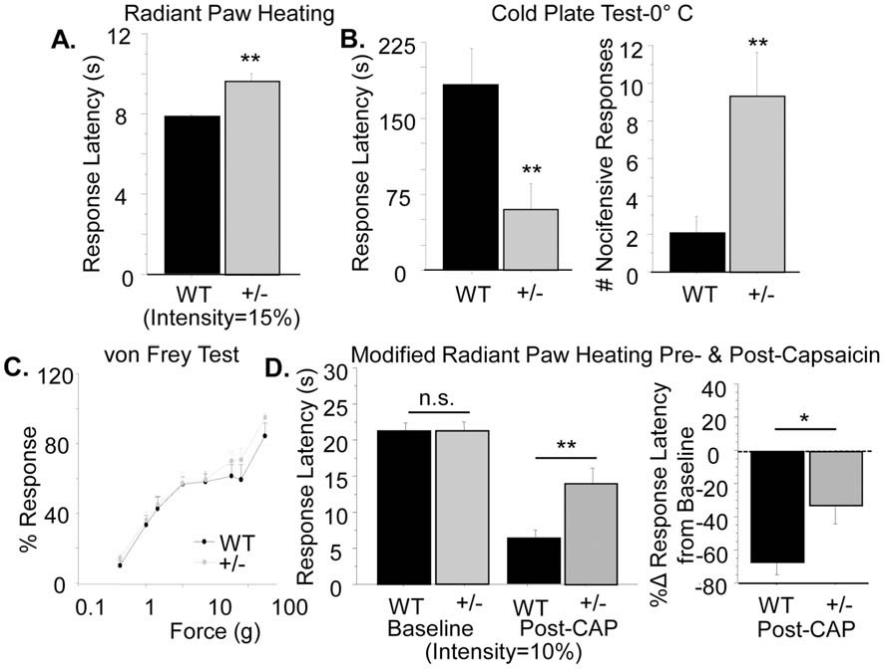
Type III Nrg1^+/−^ animals have specific behavioral deficits in responding to noxious thermal stimuli. (A) Response in radiant paw heating assay. Latency to respond to radiant heat (set at 15% of maximum intensity) applied to the hindpaw was evaluated for WT and Type III Nrg1^+/−^ siblings. Response times from both paws were averaged by animal and compared by genotype. Type III Nrg1^+/−^ mice showed a significantly increased latency to respond relative to their WT littermates (WT, n = 16 animals; Type III Nrg1^+/−^, n = 14 animals; **p<0.01). (B) Response to noxious cold assessed with the cold plate test. Latency to respond to a 0°C stimulus was measured for WT and Type III Nrg1^+/−^ mice and compared by genotype. Type III Nrg1^+/−^ mice respond significantly faster than their WT littermates (WT, n = 16 animals; Type III Nrg1^+/−^, n = 14 animals; **p<0.01). The total number of nocifensive responses that each animal made in the 5 minute period they remained on the 0°C plate was measured and compared by genotype. Type III Nrg1^+/−^ mice made significantly more nocifensive responses than their WT littermates (**p<0.01). (C) Response in the von Frey assay of mechanosensation. Percent response for the various forces was evaluated for WT and Type III Nrg1^+/−^ mice. Data were compared by genotype for each test force. There were no significant differences between genotypes (WT, n = 15 animals; Type III Nrg1^+/−^, n = 14 animals). (D) Response in a modified version of the radiant paw heating assay pre- and post-capsaicin application. Latency to respond to radiant heat (set at 10% of maximum intensity) applied to the hindpaw was evaluated for WT and Type III Nrg1^+/−^ littermates before and after application of 15 µl of 0.075% capsaicin cream to the right hindpaw. Response latencies following capsaicin application were significantly less depressed in Type III Nrg1^+/−^ animals relative to WTs (WT, n = 16 animals; Type III Nrg1^+/−^, n = 13 animals **p<0.01). Similarly, the percent change (%Δ) in response latency for Type III Nrg1^+/−^ animals was significantly blunted relative to WTs (*p<0.05). All genotype comparisons were made using a Student's t-test. All graphs show the mean±SEM.

In order to examine the effect of reducing levels of Type III Nrg1 on pain sensation we tested WT and Type III Nrg1^+/−^ adult male mice in a variety of behavioral assays that probe the ability of the animals to respond to different modalities, as well as intensities, of sensory stimuli. We assessed response to noxious heat using the Hargreaves Paw Withdrawal Assay [24]. We set the intensity of the radiant heat source (used to heat the animals' hindpaws over a thermal gradient) to a level where WT animals withdraw their paws from the stimulus at 8.01±0.21 s and found that under those parameters the Type III Nrg1^+/−^ animals respond significantly more slowly (9.45±0.39 s; p<0.01) (Figure 2A). These latencies corresponded to the time it took to heat a thermal probe to 40° and 41°C, respectively. Interesting, these temperatures are in the range of the proposed gating temperature for TRPV1 [19]. Using additional behavioral assays, we demonstrated that this deficit in Type III Nrg1^+/−^ animals was specific to noxious heat. When we assessed response to noxious cold by placing the animals on a metal plate pre-cooled to 0°C, we found that the Type III Nrg1^+/−^ animals actually generated a nocifensive response significantly faster than their WT littermates (latency to respond: WT, 183±37.0 s; Type III Nrg1^+/−^, 59.4±26.1 s; p<0.01) and generated more nocifensive responses (WT, 2±1; Type III Nrg1^+/−^, 9±2; p<0.01) in the five minute time frame in which they were kept on the plate (Figure 2B). We did not observe any significant differences between genotypes in percent response to noxious mechanical stimulation with filaments of varying intensities in the von Frey Assay (Figure 2C), further emphasizing the thermal specificity of the behavioral deficit in the Type III Nrg1^+/−^ mice. In aggregate, our studies demonstrate that Type III Nrg1^+/−^ animals have selective deficits in noxious heat sensation.

We also wanted to examine the effect of reducing levels of Type III Nrg1 on the development of inflammatory pain. To do this, we evaluated response latency in a modified version of the Hargreaves Paw Withdrawal Assay before and after the induction of inflammation in the hindpaw. We wished to increase the baseline response latency in the Hargreaves task in order to provide a larger window in which to evaluate the extent of hypersensitivity to pain developed as a result of an inflammation-inducing manipulation to one of the hindpaws. Therefore, we decreased the intensity of the radiant heat source (from 15% to 10% of maximum intensity) so that it took a longer time to elicit a withdrawal response. The new baseline response latencies for the WT and Type III Nrg^+/−^ animals were longer than what they had been (WT, 21.22±1.05 s; Type III Nrg1^+/−^, 21.20±1.35 s), and they did not differ significantly from one another (Figure 2D). At the reduced radiant heat source intensity used in these experiments, the response latencies of the animals corresponded to approximately 30°C. In the absence of inflammation, this temperature is generally not perceived as noxious. Therefore, at baseline in this experimental paradigm, it is possible that the animals were withdrawing their paws when they first perceived a warm stimulus, rather than a painful one. The different intensity of the thermal stimulus used in these experiments may also explain why we did not see any difference in response times at baseline between genotypes. Inflammation was induced by local capsaicin application to the right hindpaw, and 20 minutes later WT animals exhibited a robust reduction in response latency indicative of the development of thermal hypersensitivity to pain (WT post-capsaicin, 6.21±1.06 s; p<0.001 v. WT pre-capsaicin). Type III Nrg1^+/−^ animals also exhibited a reduction in response latency following capsaicin application (Type III Nrg1^+/−^ post-capsaicin, 13.85±2.31 s; p<0.01 v. Type III Nrg1^+/−^ pre-capsaicin), although the response latency for Type III Nrg1^+/−^ animals post-capsaicin was significantly greater than WTs (p<0.01; Figure 2D). Thus, following capsaicin application, the thermal response latency of WTs decreased by 67% whereas the response latency of the heterozygous littermates decreased by only 33% (p<0.05; Figure 2D).

### Sensory neuron survival is not affected in Type III Nrg1^+/−^ lumbar DRG

**Figure 3:**
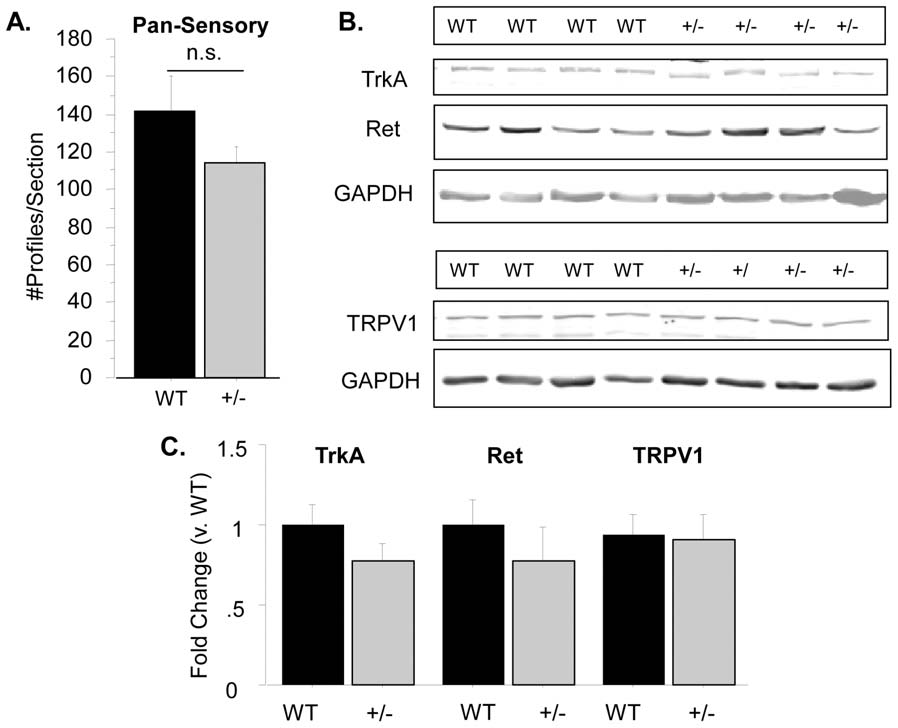
Adult WT and Type III Nrg1^+/−^ animals have equivalent numbers of sensory neurons and sensory cutaneous projections. (A) Representative sections through L4/L5 DRG from adult WT and Type III Nrg1^+/−^ mice were stained with a pan-sensory marker. Total cells staining positive for this marker tallied from at least 5 sections evenly spaced throughout the DRG were counted and averaged for each animal. Genotype averages were compared with a Student's t-test (n = 3 animals per genotype). There was no statistically significant difference between genotypes. (B) Galabrous hindpaw skin samples from WT and Type III Nrg1^+/−^ mice were assessed for total TrkA, Ret and TRPV1 protein using immunoblot. (C) The intensity of the TrkA, Ret and TRPV1 bands were quantified and normalized to GAPDH to control for equal protein loading. The values were expressed as a fold change from the average WT value and genotype averages were compared with a Student's t-test (for TrkA and Ret: WT n = 13 paws from 10 animals, Type III Nrg1^+/−^ n = 13 paws from 9 animals; for TRPV1: WT n = 9 paws from 5 animals, Type III Nrg1^+/−^ n = 8 paws from 5 animals). There was no statistically significant difference between the genotypes.

Type III Nrg1 is required for TrkA^+^ sensory neuron survival during late embryonic development [7]. Therefore, we undertook quantitative immunohistochemical analysis of L4/L5 DRG from adult WT and Type III Nrg1^+/−^ mice to assess the effect of reducing levels of Type III Nrg1 on sensory neuron survival. Sections were double-labeled with markers for sensory neuron subgroups (TrkA, IB4, NF200 and peripherin) as well as with a pan-sensory neuron marker (a cocktail of NF200 and peripherin), and the percentage of sensory neurons expressing each of the different markers was calculated. We found that reducing levels of Type III Nrg1 did not affect the percentages of any of these sensory neuron populations (TrkA: WT 30.4±1.9, Type III Nrg1^+/−^ 25.3±3.4; IB4: WT 32.1±1.9, Type III Nrg1^+/−^ 29.5±0.4; NF-200: WT 35.4±1.9, Type III Nrg1^+/−^ 33.2±2.6; Peripherin: WT 65.0±1.6, Type III Nrg1^+/−^ 66.4±2.9). Finally, the number of pan-sensory^+^ profiles per section from at least five sections evenly spaced throughout the DRG was averaged for each animal and animal averages were compared by genotype. There was no significant difference between the genotypes (Figure 3A; WT, 141±19 profiles/section; Type III Nrg1^+/−^, 114±9; p = 0.27). Thus, although Type III Nrg1 is required for sensory neuron survival, the reduced levels of Type III Nrg1 expressed in the heterozygote are sufficient to support sensory neuron survival into and throughout adulthood.

### Sensory neuron cutaneous projections are not affected in Type III Nrg1^+/−^ mice

Although we did not find any loss of sensory neuron soma in the Type III Nrg1+/− mice, it was possible that these animals had a deficit in sensory cutaneous projections. To address this possibility, we quantified TrkA, Ret and TRPV1 protein in samples of galabrous hindpaw skin from WT and Type III Nrg1+/− mice by immunoblot (Figure 3B, C). We did not see any loss of these proteins in the galabrous skin of Type III Nrg1^+/−^ mice, indicating that cutaneous projections in these animals were grossly normal.

### Type III Nrg1^+/−^ animals have a reduction in functional TRPV1 along sensory neuron axons

The capsaicin receptor, TRPV1, is a cation-permeable ion channel found in nociceptive sensory neurons that contributes to noxious temperature sensation and thermal hypersensitivity to pain under inflammatory conditions [3], [19]. We had previously verified that Type III Nrg1 and TRPV1 are found in the same sensory neuron cell bodies *in vivo* by labeling transverse sections through L4/L5 DRG from adult WT mice with antibodies against TRPV1 and Type III Nrg1 (Figure 1A(g–i)).

**Figure 4:**
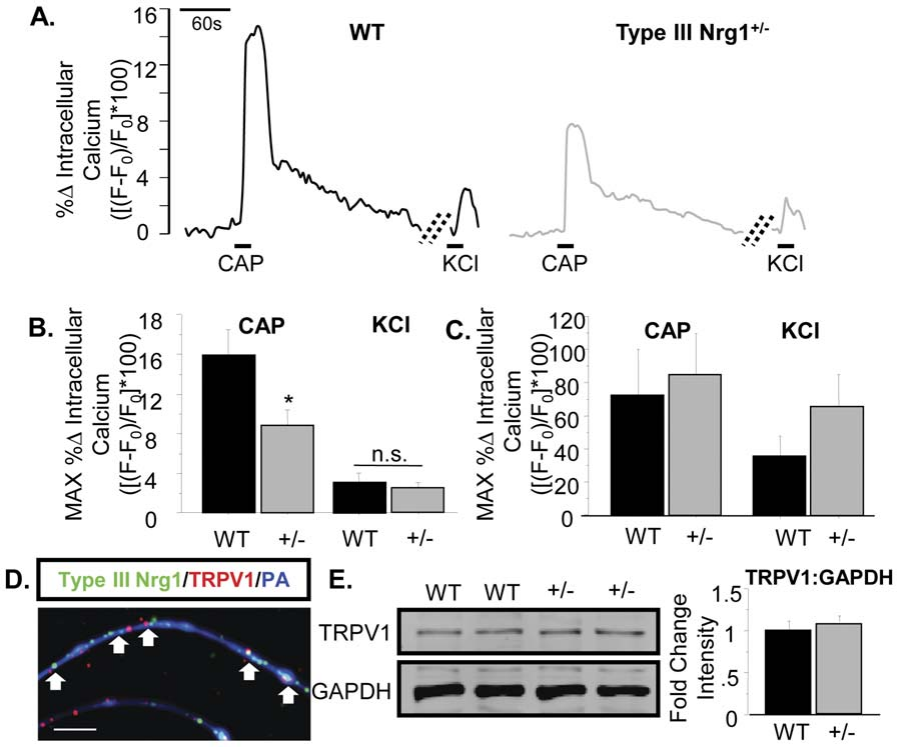
Sensory axons, but not soma, from Type III Nrg1^+/−^ mice show reduced capsaicin responsiveness compared to axons from WT mice. (A) Representative traces of intracellular calcium along sensory axons in response to 1 µM capsaicin or 56 mM KCl. The change in intracellular calcium from baseline over time ([(F−F_0_)/F_0_]*100) is shown for WT (left) and Type III Nrg1^+/−^ (right) axons. Hatched diagonal lines indicate where the time course was non-continuous. (B) Quantification of the maximum change in intracellular calcium in response to application of 1 µM capsaicin or 56 mM KCl by genotype. Averages of 5 animals per genotype were compared using a Student's t-test. Type III Nrg1^+/−^ axons showed a significantly decreased response to capsaicin (p<0.05), but not to KCl, relative to WTs. Graph shows mean±SEM. (C) Type III Nrg1^+/−^ sensory soma show normal response to capsaicin. Quantification of maximal change in fluorescence from baseline ([(F−F_0_)/F_0_]*100) in WT or Type III Nrg1^+/−^ sensory neuron *soma* in response to 1 µM capsaicin or 56 mM KCl. Average responses from 4 WT and 4 Type III Nrg1^+/−^ animals to application of capsaicin or KCl were compared by genotype using a Student's t-test. There was no statistically significant difference between genotypes. Graphs show mean±SEM. (D) Type III Nrg1 (green) and TRPV1 (red) are co-expressed along P21 WT cultured sensory neuron axons identified with a pan-axonal (PA) marker (blue). White arrows indicate examples where Type III Nrg1 and TRPV1 are in close proximity. Scale bar equals 10 µm. (E) P21 WT and Type III Nrg1^+/−^ sensory neuron cultures have equivalent levels of total TRPV1 protein. Total TRPV1 protein measurement by immunoblot. The 95 kD TRPV1 band and the 35 kD GAPDH band are shown from a representative experiment comparing protein from P21 WT and Type III Nrg1^+/−^ cultures. Quantification of fold change in intensity of TRPV1∶GAPDH normalized to WT average. There was no statistically significant change in the ratio of TRPV1 to GAPDH between genotypes (WT, Type III Nrg1^+/−^, n = 3 animals). Genotype comparisons were made using a Student's t-test. Graph shows mean±SEM.

TRPV1 located along axons and at peripheral terminals is most physiologically relevant to temperature and pain sensation. We verified that Type III Nrg1 and TRPV1 were co-expressed in the same sensory neuron axons by labeling sections from the plantar hindpaw skin of adult WT mice with antibodies against Type III Nrg1 and TRPV1 (Figure 1B (d′–f′)). Additionally, Type III Nrg1 and TRPV1 are co-expressed *in vitro* along the axons of sensory neurons from P21 WT male mice (Figure 4D).

As TRPV1 is permeable to calcium, we measured functional TRPV1 along sensory axons by quantifying agonist-induced changes in intracellular calcium. For twenty seconds, 1 µM capsaicin was focally applied to cultures of DRG sensory neurons from P21 WT male mice. Capsaicin treatment resulted in a 16 percent increase in intracellular calcium from baseline ([(F−F_0_)/F_0_]*100) (Figure 4). This response was eliminated by perfusion with 10 µM capsazapine, a selective TRPV1 antagonist (data not shown). In addition, no response to capsaicin was seen when similar experiments were done using cultures of DRG sensory neurons from TRPV1^−/−^ mice (data not shown). Next, we compared the response to capsaicin along WT axons to that seen along sensory axons from Type III Nrg1^+/−^ mice. Axons from Type III Nrg1^+/−^ mice had a significantly reduced maximum change in fluorescence in response to focal application of 1 µM capsaicin compared to WT axons (WT, 15.94±2.61%; Type III Nrg1^+/−^, 8.82±1.63%; p<0.05) (Figure 4B). Interestingly, there was not a significant difference between the genotypes when comparing the maximum *somal* response to capsaicin (Figure 4C; WT, 72.3±27.5%; Type III Nrg1^+/−^, 84.8±25.3%; p = 0.75). Finally, there was no effect of genotype on the maximum change in fluorescence in response to depolarization with 56 mM KCl either along axons (Figure 4B; WT, 3.09±0.95%; Type III Nrg1^+/−^, 2.50±0.56%; p = 0.63) or within soma (Figure 4C, WT, 35.8±26.3%; Type III Nrg1^+/−^, 66.4±37.7%; p = 0.23), indicating that the decreased calcium signal in response to capsaicin seen in Type III Nrg1^+/−^ axons was not the result of changes in voltage gated calcium or tetrodotoxin (TTX)-resistant sodium channels. Cumulatively, these data show that the TRPV1 deficit in Type III Nrg1^+/−^ sensory neurons appears to be localized to axons, and does not appear to be indicative of abnormalities in other axonally-localized calcium-permeable ion channels. This deficit in functional TRPV1 along the axons of Type III Nrg1^+/−^ sensory neurons is consistent with the reduced capsaicin-induced thermal hypersensitivity to pain seen in these animals.

### Acute activation of Type III Nrg1 back signaling enhances levels of functional TRPV1 receptors along WT sensory neuron axons

Type III Nrg1 is a bi-directional signaling molecule. While Type III Nrg1 acting as a ligand can stimulate ErbB receptor tyrosine kinase signaling, Type III Nrg1 signaling as a receptor can be activated by interaction with ErbB receptor tyrosine kinases or by depolarization [1], [22]. As Type III Nrg1 and TRPV1 are expressed in the same cell, we hypothesized that Type III Nrg1 was affecting TRPV1 by signaling as a receptor. Type III Nrg1 signaling as a receptor (Type III Nrg1 back signaling) can exert both transcription-dependent and transcription-independent effects [1], [2], [22], [23]. To evaluate whether the deficit in functional TRPV1 along Type III Nrg1^+/−^ axons was due to transcription-dependent or transcription-independent effects of reducing levels of Type III Nrg1, we compared total TRPV1 protein in cultures from P21 male WT and Type III Nrg1^+/−^ mice (Figure 4E). As we found no difference in total TRPV1 protein between the genotypes, we concluded the effect of Type III Nrg1 signaling on functional TRPV1 along axons was most likely to be transcription-independent.

**Figure 5:**
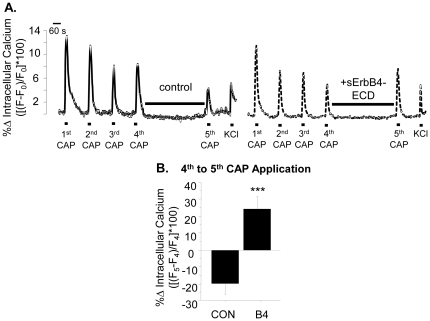
Stimulation of Type III Nrg1 signaling enhances response to capsaicin in WT sensory axons. (A) Representative traces of intracellular calcium along WT sensory axons in response to repeated applications of 1 µM capsaicin followed by application of 56 mM KCl. The maximum percent change in intracellular calcium ([(F−F_0_)/F_0_]*100) in response to capsaicin decreased between the 4^th^ and 5^th^ capsaicin applications under control conditions (left), but increased when Type III Nrg1 signaling was stimulated by sErbB4-ECD application during that interval (right). (B) Quantification of percent change in maximum response to capsaicin between the 4^th^ and the 5^th^ capsaicin application ([(F_5_−F_4_)/F_4_]*100) by treatment. WT sensory axons showed a significantly enhanced response to capsaicin when Type III Nrg1 signaling was stimulated with sErbB4-ECD (WT CON, n = 10 animals; WT B4, n = 7 animals; Student's t-test, ***p<0.001). Graph shows mean±SEM.

To test whether Type III Nrg1 back signaling acutely affects levels of functional TRPV1 along sensory neuron axons, we used a calcium imaging paradigm with multiple applications of capsaicin where the fourth and fifth capsaicin applications were separated by a 12 minute rest period during which Type III Nrg1 back signaling could be acutely stimulated (Figure 5A). Repeated applications of capsaicin cause progressively smaller responses, most likely due to calcium dependent desensitization of TRPV1 channels [25]. When we treated WT sensory axons with 4 capsaicin applications spaced 4 minutes apart there was a progressive decrease in responsiveness. Response to a 5^th^ capsaicin application after a 12 minute rest period was on average 19.78±5.71% smaller than the response to the 4^th^ capsaicin application (Figure 5B). To stimulate Type III Nrg1 back signaling, we applied a solubilized form of the extracellular domain of the ErbB4 receptor tyrosine kinase (sErbB4-ECD). Stimulation of Type III Nrg1 back signaling during this 12 minute interval reversed the decrease in capsaicin responsiveness, and in fact increased the amplitude by 24.11±8.07% (Figure 5B). This increase in capsaicin responsiveness following stimulation of Type III Nrg1 back signaling was significantly greater than the decrease seen under control conditions (p<0.001). Importantly, stimulation of Type III Nrg1 back signaling did not affect response to depolarization with KCl (data not shown), indicating that the target of Type III Nrg1 back signaling was TRPV1 and not voltage-gated calcium or TTX-resistant sodium channels. Thus, acutely stimulating Type III Nrg1 back signaling along WT sensory axons enhances functional *axonal* TRPV1.

### PtdIns3K mediates signaling between Type III Nrg1 and TRPV1

**Figure 6:**
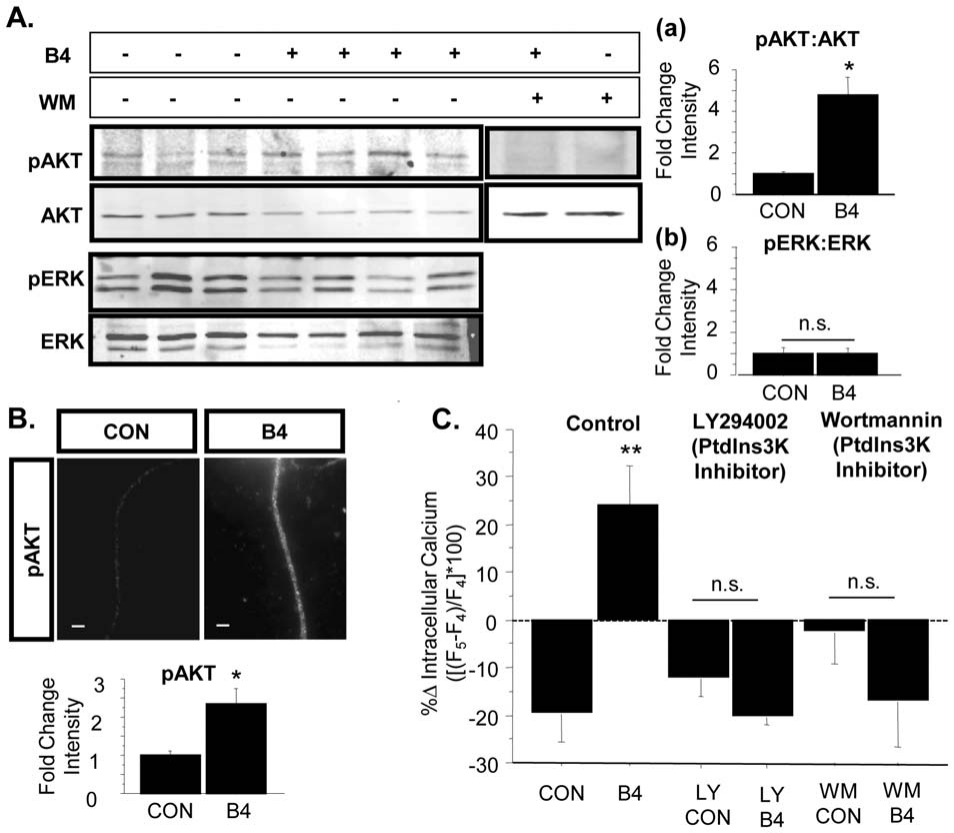
PtdIns3K activation is required for Type III Nrg1-induced enhancement of capsaicin responsiveness. (A) 15-minute stimulation of Type III Nrg1 signaling with sErbB4-ECD significantly increased the ratio of pAKT-AKT in whole cell lysates from P21 WT sensory neuron cultures (a: CON, n = 3; B4, n = 4; Student's t-test, *p<0.05). This increase was blocked by pre-incubation with 20 nM wortmannin (WM), an inhibitor of PtdIns3K activity. (b) The same stimulation of Type III Nrg1 with sErbB4-ECD did not activate the MAPK pathway as illustrated by the lack of effect on the ratio of pERK-ERK. (B) Stimulation of Type III Nrg1 signaling with sErbB4-ECD significantly increased average fluorescence intensity (AFI) levels for pAKT staining along P21 WT cultured sensory axons (15 minutes; CON, n = 3; B4, n = 3; Student's t-test, *p<0.05). Scale bars equal 10 µm. (C) Blocking PtdIns3K signaling with 50 µM LY294002 or 20 nM wortmannin blocked the Type III Nrg1-induced enhancement of functional TRPV1 (LY CON, n = 3 animals; LY B4, n = 2; WM CON, n = 10; WM B4, n = 7). Comparisons between CON and all treatment groups were made using an ANOVA with a Holm-Sidak post-hoc test for multiple comparisons. All graphs show mean±SEM.

Levels of functional, surface TRPV1 in sensory neuron soma or HEK-293 cells are known to be modulated by a number of signaling cascades including PtdIns3K [4], [5], [6]. Type III Nrg1 back signaling also activates PtdIns3K in cultures from P21 WT sensory neurons (Figure 6A, B). 15-minute stimulation of Type III Nrg1 signaling with sErbB4-ECD resulted in an increase in the ratio of pAKT-AKT in whole cell lysates from P21 WT sensory neuron cultures, as assayed by immunoblot (Figure 6A(a); WT B4, 4.8±0.9-fold increase from WT CON; p<0.05). This effect of Type III Nrg1 signaling as a receptor is pathway specific as the same stimulation paradigm did not change the ratio of pERK-ERK (Figure 6A(b); p = 0.98). Additionally, 15-minute stimulation with sErbB4-ECD increased the fluorescence intensity of pAKT staining along axons of cultured sensory neurons from P21 WTs (Figure 6B; WT B4, 2.3±0.4-fold increase from WT CON; p<0.05), consistent with Type III Nrg1 activation of PtdIns3K signaling along sensory axons. We then investigated whether PtdIns3K activation mediates signaling between Type III Nrg1 and TRPV1. When PtdIns3K signaling was blocked by bath application of 50 µM LY294002 or 20 nM wortmannin during calcium imaging, there was no effect on baseline response to capsaicin (data not shown), but the effect of stimulating Type III Nrg1 back signaling on capsaicin responsiveness was abolished (6C; WT LY CON, −12.2±3.7% decrease between 4^th^ and 5^th^ capsaicin applications; WT LY B4, −20.0±1.7%; WT WM CON, −2.5±6.7%; WT WM B4, −17.4±9.5%).

### Pre-incubation with sErbB4-ECD enhances response to the second, but not the first, application of capsaicin along sensory axons

**Figure 7:**
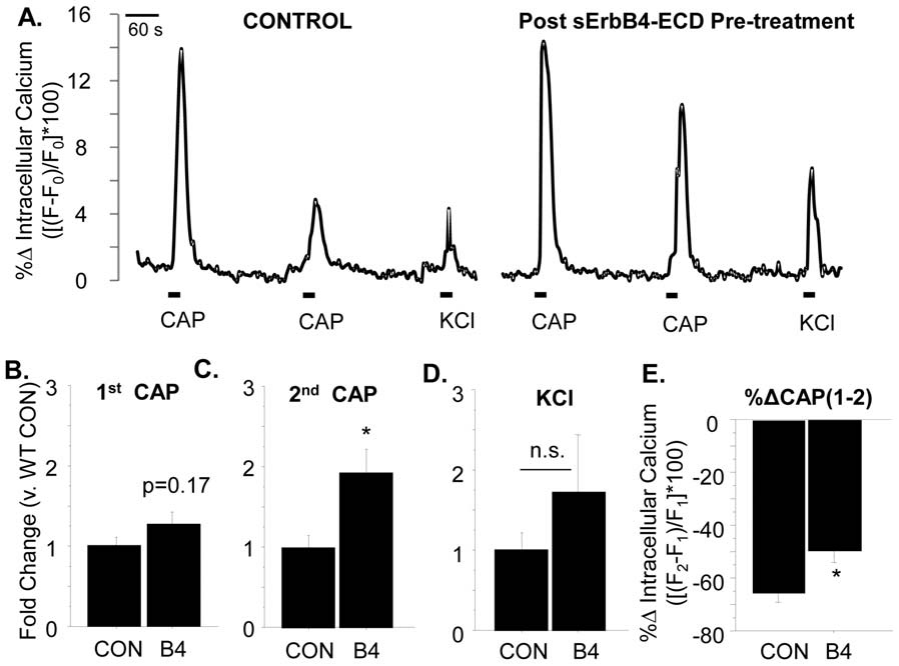
Pre-treatment with sErbB4-ECD enhances response to a second application of capsaicin along sensory axons. (A) Sensory neuron cultures from P21 WT mice were incubated with sErbB4-ECD or control media for 12 minutes before two 1 µM pulses of capsaicin were applied (spaced 4 minutes apart), followed by application of 56 mM KCl. Within axons that responded to both capsaicin and KCl, the percent change in fluorescence from baseline ([(F−F_0_)/F_0_]*100) in response to both applications of capsaicin as well as KCl was calculated. Axonal responses were averaged by animal and normalized to the WT CON average. (B–C) Quantification of the maximum response to the first (B) or second (C) applications of 1 µM capsaicin were compared by treatment. Pre-treatment with sErbB4-ECD significantly increased the maximum response to the second pulse of capsaicin (WT CON, n = 8 animals; WT B4, n = 9 animals; *p<0.05). There was a trend towards an increase in response to the first pulse of capsaicin following sErbB4-ECD pre-treatment (p = 0.17). (D) Quantification of the maximum response to KCl by treatment. sErbB4-ECD pre-treatment had no effect on the maximum response to KCl (p = 0.38). (E) The percent change between the maximum response to the first and second capsaicin applications was calculated and axonal values were averaged by animal. Quantification of this percent change in response by treatment; Cultures pre-treated with sErbB4-ECD showed significantly less of a decrease in responsiveness to capsaicin between the first and second capsaicin applications (*p<0.05). All treatment comparisons were made using a Student's t-test. Graphs show mean±SEM.

The enhancement of functional TRPV1 seen following acute stimulation of Type III Nrg1 back signaling in WT sensory axons could be due to either insertion of new TRPV1 receptors or modifications of pre-existing receptors that alter channel functionality. To distinguish between these possibilities we pre-treated cultures with sErbB4-ECD for 12 minutes and then applied 2 pulses of capsaicin spaced 4 minutes apart, reasoning that the channels should be in a relatively un-desensitized state when we applied the first pulse of capsaicin, but not when we applied the second one. If stimulation of Type III Nrg1 back signaling increased the number of functional TRPV1 receptors on the axonal membrane, we would expect to see an increase in response to the first application of capsaicin in cultures pre-treated with sErbB4-ECD. In fact, stimulating Type III Nrg1 signaling resulted in a slight, not significant increase in response to the first capsaicin application (Figure 7B; WT B4, 1.27-fold increase, p = 0.17). However, if stimulation of Type III Nrg1 signaling affected receptor desensitization, we would expect to see an increase in the response to the second application of capsaicin (when the receptors are in a desensitized state) in cultures pre-treated with sErbB4-ECD. Indeed, the amplitude of the response to the second application of capsaicin was significantly greater in cultures where Type III Nrg1 signaling as a receptor had been stimulated by pre-incubation with sErbB4-ECD (Figure 7C; WT B4, 1.93-fold increase, p<0.05). In both control and sErbB4-ECD treated cultures the response to the second application of capsaicin was smaller than the first, most likely due to calcium-dependent tachyphalaxsis. In control cultures, there was on average a 65.66±3.36% decrease in responsiveness between the first and second capsaicin applications (Figure 7E). However, in cultures stimulated with sErbB4-ECD this decrease was reduced to 49.67±4.30%, a statistically significant difference from control conditions (Figure 7E; p<0.05). There was no effect of pre-stimulation with sErbB4-ECD on responsiveness to depolarization with KCl (p = 0.38; Figure 7D). Therefore, the major effect of stimulating Type III Nrg1 back signaling would appear to be a change in the degree of desensitization.

### sErbB4-ECD stimulation does not affect functional TRPV1 by blocking signaling between endogenous Nrg1 and ErbB receptors

**Figure 8:**
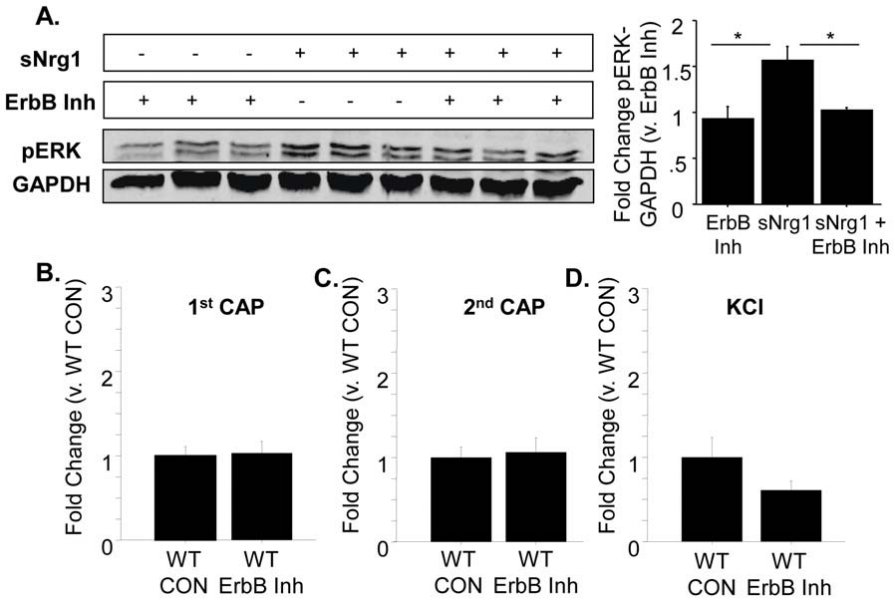
Blockade of ErbB signaling does not enhance response to capsaicin in WT sensory axons. (A) Incubation of P21 WT sensory neuron cultures with soluble Nrg1 (sNrg1) stimulates ErbB signaling and activates ERK (measured as a ratio of pERK to GAPDH). This sNrg1-induced activation of ERK can be blocked by incubation with 1 µM PD158780, an ErbB kinase inhibitor (n = 3 animals per condition; *p<0.05). (B–D) Blockade of ErbB signaling does not enhance response to application of capsaicin or KCl. Sensory neuron cultures from P21 WT mice were incubated with 1 µM PD158780 or control media for 12 minutes before two 1 µM pulses of capsaicin were applied (spaced 4 minutes apart), followed by application of 56 mM KCl. Within axons that responded to both capsaicin and KCl, the percent change in fluorescence from baseline ([(F−F_0_)/F_0_]*100) in response to both applications of capsaicin as well as KCl was calculated. Axonal responses were averaged by animal and normalized to the WT CON average. Quantification of the maximum response to the first (B) or second (C) applications of 1 µM capsaicin or KCl (D) were compared by treatment. Blockade of ErbB signaling with 1 µM PD158780 did not affect response to the first or second application of capsaicin, or to KCl (WT CON, n = 7 animals; ErbB Inh, n = 7 animals).

It was possible that all the effects of applying sErbB4-ECD on capsaicin responsiveness were due to blockade of endogenous Nrg1 stimulation of ErbB3 receptors located on adjacent Schwann cells, rather than to direct stimulation of Type III Nrg1 back signaling. If this alternative scenario were true then endogenous ErbB receptor-mediated signaling should be tonically depressing levels of functional TRPV1. Thus, blocking ErbB receptor signaling should disinihibit this inhibition and enhance levels of functional TRPV1. To test this possibility, we decided to block ErbB receptor-mediated signaling by applying the ErbB kinase inhibitor, PD158780. At 1 µM, this drug completely blocked ErbB receptor-mediated signaling, as indicated by its ability to block soluble Nrg1 (sNrg1)-induced stimulation of ErbB receptors and activation of ERK (Figure 8A). We then incubated our sensory neuron cultures in this concentration of ErbB receptor inhibitor for 15 minutes prior to applying 2 pulses of capsaicin, reasoning that if sErbB4-ECD was exerting its effects by blocking endogenous ErbB receptor signaling then directly blocking ErbB receptor signaling should mimic the effect of sErbB4-ECD application. Instead, we found that blockade of ErbB receptor signaling had no effect on subsequent capsaicin responsiveness (Figure 8B & C). This result provided further evidence that acutely stimulating Type III Nrg1 back signaling along WT sensory axons enhances functional *axonal* TRPV1.

### Stimulation of Type III Nrg1 back signaling with sErbB4-ECD fails to rescue deficits in functional TRPV1 along Type III Nrg1^+/−^ sensory axons

**Figure 9:**
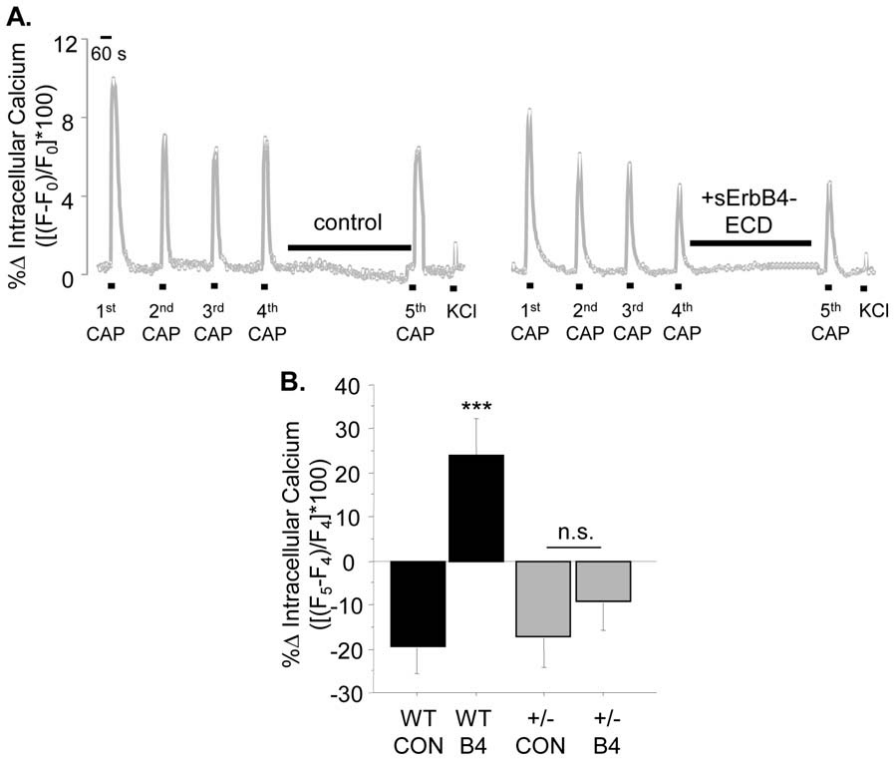
Acute stimulation of Type III Nrg1 signaling in Type III Nrg1^+/−^ sensory axons does not rescue functional TRPV1 deficits. (A) Representative traces of intracellular calcium along Type III Nrg1^+/−^ sensory axons in response to repeated applications of 1 µM capsaicin followed by application of 56 mM KCl. The maximum percent change in intracellular calcium ([(F−F_0_)/F_0_]*100) in response to capsaicin decreased between the 4^th^ and 5^th^ capsaicin application in Type III Nrg1^+/−^ axons under control conditions (left) and did not increase when Type III Nrg1 signaling was stimulated by sErbB4-ECD application during that interval (right). (B) Quantification of percent change in maximum response to capsaicin between the 4^th^ and the 5^th^ capsaicin application ([(F_5_−F_4_)/F_4_]*100) by treatment. Results from WT sensory axons are included for comparison (WT CON, n = 10; WT B4, n = 7; ***p<0.001). Type III Nrg1^+/−^ axons did not show a statistically significantly enhanced response to capsaicin when Type III Nrg1 signaling was stimulated (Type III Nrg1^+/−^ CON, n = 6 animals; Type III Nrg1^+/−^ B4, n = 8). All comparisons between genotypes and treatments were made using an ANOVA with a Holm-Sidak post-hoc test for multiple comparisons. Graph shows mean±SEM.

As acute stimulation of Type III Nrg1 back signaling enhances functional TRPV1 along sensory axons, and Type III Nrg1^+/−^ sensory axons have reduced levels of TRPV1, we attempted to rescue levels of functional TRPV1 along Type III Nrg1^+/−^ axons by acutely stimulating Type III Nrg1^+/−^ sensory neuron cultures with sErbB4-ECD. Using the capsaicin application paradigm described above (for Figure 5), we found that Type III Nrg1^+/−^ sensory axons showed the same degree of desensitization to repeated capsaicin applications as WT axons. In Type III Nrg1^+/−^ sensory axons a 5^th^ capsaicin application after a 12 minute rest period was on average 17.6±6.4% smaller than the response to the 4^th^ capsaicin application, which was not significantly different than the 19.78±5.71% decrease seen in WT axons (p = 0.8; Figure 9B). Unexpectedly, we found that acute stimulation of Type III Nrg1 back signaling along Type III Nrg1^+/−^ sensory axons during this 12 minute interval did not enhance the response to capsaicin as was seen in WT axons (Figure 9B; Type III Nrg1^+/−^ B4, −9.6±6.21%; for reference, WT B4, +24.11±8.07%). Thus, sensory axons with reduced Type III Nrg1 were deficient in both basal levels of functional TRPV1 and in the ability to acutely up-regulate functional TRPV1 in response to exogenous stimulation of Type III Nrg1 back signaling.

### Stimulation of Type III Nrg1 back signaling with sErbB4-ECD does not activate PtdIns3K signaling along Type III Nrg1^+/−^ sensory axons

**Figure 10:**
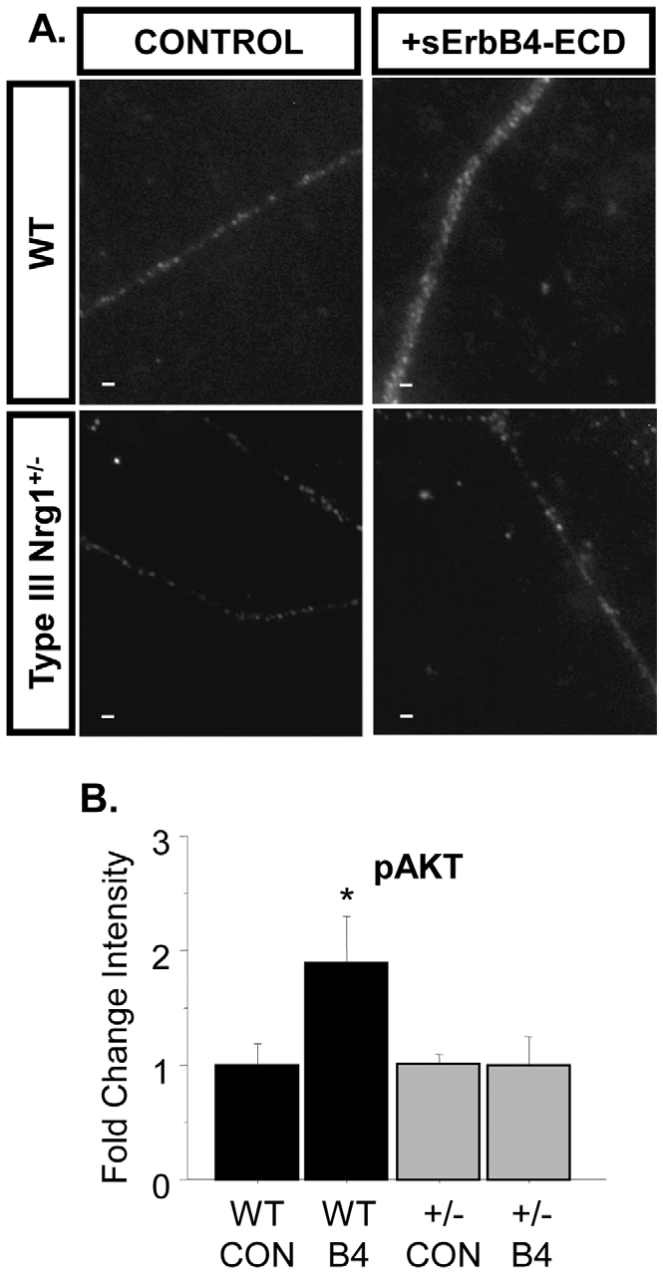
sErbB4-ECD stimulation of Type III Nrg1^+/−^ sensory neuron cultures fails to activate PtdIns3K along sensory axons. (A) Representative images of pAKT staining along WT and Type III Nrg1^+/−^ sensory axons stimulated for 15 minutes with either control or sErbB4-ECD media. All scale bars equal 10 µm. (B) Quantification of pAKT average fluorescence intensity (AFI) along WT and Type III Nrg1^+/−^ sensory axons under various treatment conditions. sErbB4-ECD stimulation significantly increased AFI levels for pAKT staining along P21 WT sensory axons (WT CON, n = 3 animals; WT B4, n = 3; *p<0.05) but not along sensory axons from Type III Nrg1^+/−^ cultures (Type III Nrg1^+/−^ CON, n = 3 animals; Type III Nrg1^+/−^ B4, n = 3; p = 0.9). All comparisons between treatments and genotypes were made using an ANOVA with a Fischer's PLSD post-hoc analysis. Graph shows mean±SEM.

Axons from Type III Nrg1^+/−^ animals fail to up-regulate functional TRPV1 in response to stimulation of Type III Nrg1 back signaling. We wondered if this impairment resulted from an inability to activate PtdIns3K signaling. To test this, we stimulated P21 WT and Type III Nrg1^+/−^ cultures with sErbB4-ECD for 15 minutes and then quantified the intensity of pAKT staining along sensory axons. When stimulated with sErbB4-ECD, cultures from WT animals showed a significant increase in axonal pAKT (1.9-fold increase; p<0.05), but cultures from Type III Nrg1^+/−^ animals did not (p = 0.97) (Figure 10B). Both P21 WT and Type III Nrg1^+/−^ sensory neurons showed a robust activation of PtdIns3K signaling in response to a five-minute stimulation with 100 ng/ml NGF (data not shown; WT NGF, 1.8±0.28-fold change from WT CON; p<0.05; Type III Nrg1^+/−^ NGF, 2.25±0.54-fold change from WT CON; p<0.05). Thus, the deficit in PtdIns3K activation observed in Type III Nrg1^+/−^ sensory axons was specific to stimulation of Type III Nrg1 back signaling. That exogenous stimulation of Type III Nrg1 back signaling along Type III Nrg1^+/−^ sensory axons neither activated PtdIns3K, nor enhanced functional TRPV1, is consistent with the idea that a Type III Nrg1-activated PtdIns3K signal is required to enhance functional TRPV1 in WT sensory axons.

**Figure 11:**
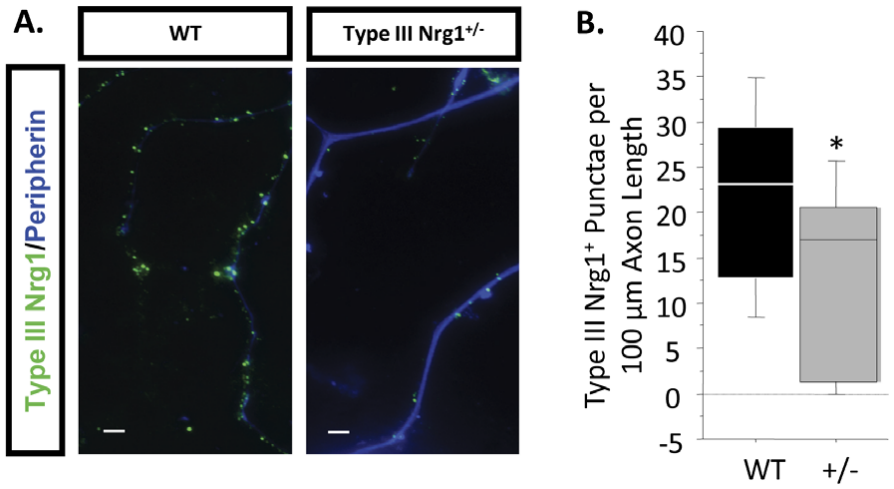
Type III Nrg1^+/−^ sensory axons have reduced numbers of Type III Nrg1^+^ punctae relative to WT sensory axons. (A) Conventional microscopic images of Type III Nrg1^+^ punctae (green) found along peripherin^+^ axons (blue) from WT and Type III Nrg1^+/−^ sensory cultures. Scale bars equal 10 µm. (B) Quantification of the average number of punctae per 100 µm axon length from 16 WT and 15 Type III Nrg1^+/−^ images. Comparison by genotype illustrates that Type III Nrg1^+/−^ sensory axons have significantly fewer Type III Nrg1^+^ punctae than WT sensory axons (Mann-Whitney Rank Sum test, *p<0.05).

**Figure 12:**
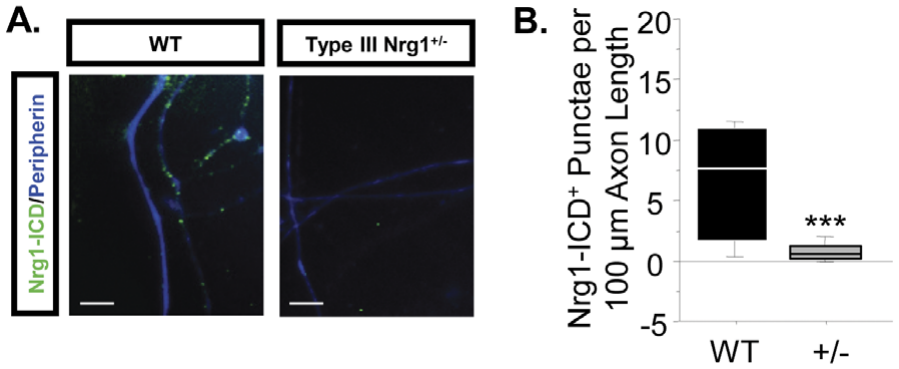
Type III Nrg1^+/−^ sensory axons have reduced numbers of Nrg1^+^ punctae that contain a signaling-competent intracellular domain (Nrg1-ICD) relative to WT sensory axons. (A) Conventional microscopic images of Nrg1-ICD^+^ punctae (green) found along peripherin^+^ axons (blue) from WT and Type III Nrg1^+/−^ sensory cultures. Scale bars equal 10 µm. (B) Quantification of the average number of punctae per 100 µm axon length from 14 WT and 15 Type III Nrg1^+/−^ images. Comparison by genotype illustrates that Type III Nrg1^+/−^ sensory axons have significantly fewer Nrg1-ICD^+^ punctae than WT sensory axons (Mann-Whitney Rank Sum test, ***p<0.001).

The absence of sErbB4-ECD-stimulated signaling in Type III Nrg1^+/−^ sensory neuron axons was unexpected. To determine if the heterozygous sensory neurons lacked axonal Type III Nrg1 we examined Type III Nrg1 expression along sensory axons using antibodies that specifically recognize Type III Nrg1 or the Nrg1-ICD. In cultures from P21 mice, Type III Nrg1 immunofluorescent staining was punctate along WT and Type III Nrg1^+/−^ sensory neuron axons identified with the nociceptive-specific marker, peripherin (Figure 11). Quantification of the number of Type III Nrg1^+^ punctae per 100 µm of sensory axon demonstrated that the number of Type III Nrg1^+^ punctae was reduced by fifty percent along Type III Nrg1^+/−^ axons (Figure 11B; WT, 22±3 puncta/100 µm; Type III Nrg1^+/−^, 13±3 puncta/100 µm; p<0.05). We found an even greater decrease in expression of Nrg1 proteins containing the ICD domain along Type III Nrg1^+/−^ sensory neuron axons relative to WT axons (Figure 12B; WT, 6±1 puncta/100 µm; Type III Nrg1^+/−^, 1±0.3 puncta/100 µm; p = 0.001). Therefore, although total levels of axonal Type III Nrg1 were reduced 2-fold in the heterozygotes, there was a 6-fold decrease in the level of signaling-competent *axonal* Type III Nrg1 in the heterozygotes. The preferential decrease in axonal Nrg1-ICD is likely to result from changes in the dynamics of proteolytic processing and targeting to axons, rather than altered splicing of TMc containing transcripts. In both WT and Type III Nrg1^+/−^ DRG ∼90% of transcripts contained the TMc domain encoding exon, whereas less than 10% utilized the β3 splice choice that would result in expression of just the Type III Nrg1β3 isoform (data not shown). It is likely that the reduced level of Type III Nrg1 ICD-containing protein along Type III Nrg1^+/−^ sensory axons was insufficient to activate PtdIns3K or regulate functional TRPV1.

## Discussion

Type III Nrg1 is involved in the perception of noxious temperatures, particularly under acute inflammatory conditions. Type III Nrg1^+/−^ mice display deficits in their response to noxious heat, and in their ability to develop thermal hypersensitivity to pain following capsaicin-induced inflammation of the hindpaw. Consistent with this behavioral phenotype we show that Type III Nrg1 is involved in the regulation of functional TRPV1 along sensory neuron axons. Type III Nrg1^+/−^ mice have reduced functional TRPV1 along their sensory axons. Additionally, acute stimulation of Type III Nrg1 back signaling in WT sensory axons enhances functional TRPV1 in a manner that is dependent on PtdIns3K activation. Acute stimulation of Type III Nrg1 back signaling in Type III Nrg1^+/−^ sensory axons, however, does not enhance functional TRPV1 or activate PtdIns3K, consistent with their blunted capsaicin induced thermal hypersensitivity. Together, these data provide support for a model whereby Type III Nrg1 back signaling in nociceptive sensory neurons helps regulate functional TRPV1 along sensory axons, thereby contributing to the ability to sense heat and to develop thermal hypersensitivity to pain under inflammatory conditions.

Type III Nrg1^+/−^ animals have hypomyelinated peripheral nerves, disorganized Remak bundles and reduced conduction velocities [26], [27]. Despite these general effects on peripheral nerves, the Type III Nrg1+/− animals have limited impairments in their response to noxious heat, an enhanced response to noxious cold and normal responses to mechanical stimuli. Over-expression of a dominant negative form of ErbB4 (DN-ErbB4) in non-myelinating Schwann cells results in decreased response to both noxious hot and cold stimuli that was attributed to a progressive loss of C-fiber sensory neurons [28]. These differences between our mice and the DN-ErbB4 mice indicate that the behavioral phenotype in the Type III Nrg1^+/−^ animals cannot be attributed to a loss of stimulation of Schwann cell ErbB receptors. Thus although altered Schwann cell function might contribute to the phenotype reported here, additional aspects of Type III Nrg1 signaling are likely to account for the sensory deficits.

Type III Nrg1 heterozygotes also differ from mice in which all Nrg1 expression is disrupted specifically in a population of small-diameter C-fiber nociceptive sensory neurons that express the sodium channel, Nav1.8 [29]. These Nav1.8-specific Nrg1^−/−^ animals showed no changes in response to thermal or mechanical stimuli, except for a deficit in response to tail pinch. Nav1.8 is not expressed in a population of peptidergic, TRPV1^+^ sensory neurons that are essential for heat pain sensation [30], [31]. Thus, the most parsimonious explanation for the difference between our findings and those of Fricker et al [29] is that Type III Nrg1 signaling in TRPV1^+^/Nav1.8^−^ nociceptive sensory neurons, contributes to heat pain sensation.

Although the present study clearly shows an effect of Type III Nrg1 signaling on TRPV1, it is likely that Type III Nrg1 affects additional aspects of sensory neuron function that contribute to the sensory behavioral deficits seen. In fact, previous results from our laboratory demonstrate that Type III Nrg1 back signaling regulates functional surface α7* nAChRs [2], [32], indicating that Type III Nrg1 modulates multiple ion channels that contribute to temperature and pain sensation. Type III Nrg1 is also expressed in other neurons that contribute to response to painful stimuli and peripheral nerves in the Type III Nrg1 heterozygotes are hypomyelinated with altered conduction velocities [26], [27]. While we cannot rule out a contribution of hypomyelination or Type III Nrg1 signaling in other areas of the nervous system to the phenotype in the Type III Nrg1^+/−^ mice, the modality specific nature of the behavioral deficits in the Type III Nrg1^+/−^ mouse is consistent with a deficiency in Type III Nrg1 signaling in a specific population of sensory neurons.

Consistent with our behavioral results, we present evidence that Type III Nrg1 acting along sensory axons can regulate levels of functional TRPV1. Under inflammatory conditions, rapid activation of a variety of signaling pathways can acutely increase TRPV1 levels on the surface of sensory neurons, potentiate TRPV1 channel function and decrease the threshold for TRPV1 channel activation [33], [34]. All of these mechanisms render sensory neurons more responsive to subsequent thermal stimulation, notably at temperatures at which they would have previously remained quiescent. We demonstrate that acute stimulation of Type III Nrg1 back signaling rapidly increases functional TRPV1 along cultured WT sensory neuron axons in a PtdIns3K-dependent manner. Previous studies linking PtdIns3K activation by NGF-TrkA signaling to acute insertion of new TRPV1 receptors into the cell membrane [4], [5], [6] relied on measurements made in either the soma of cultured mouse sensory neurons or heterologous expression systems. It was assumed that an analogous mechanism occurs along sensory axons and at peripheral nerve terminals, where this signaling would matter *in vivo*. Our data provide evidence that a PtdIns3K-dependent enhancement of TRPV1 can also occur along sensory axons *in vitro*. Furthermore, we show that Type III Nrg1 is present along TRPV1^+^ peripheral nerve terminals innervating hindpaw skin, providing plausibility for our mechanism to occur in a physiologically relevant context *in vivo*.

Stimulating Type III Nrg1 signaling in Type III Nrg1^+/−^ sensory neuron cultures had no effect on either functional axonal TRPV1 or PtdIns3K activation. Immunofluorescent detection of Type III Nrg1 and Nrg1-ICD protein revealed that Type III Nrg1^+/−^ animals have a two- and six-fold decrease in the number of Type III Nrg1^+^ and Nrg1-ICD^+^ punctae along their sensory axons, respectively, as compared to WTs. Therefore, it is likely that a threshold level of signaling-competent, ICD-containing Type III Nrg1 protein along axons is needed to achieve detectable effects on TRPV1 and PtdIns3K. These results also demonstrate that in vitro, there are two pools of Type III Nrg1 along nociceptive axons: one that is competent to engage in bi-directional signaling, the other that is not. In vivo, the latter pool is likely to provide the axonal Type III Nrg1 required by Schwann cells to organize axons into Remak bundles [27]. RT-PCR analyses of Type III Nrg1 expression in mouse DRG indicates that the majority of transcripts in both wild type and heterozygotes encode Type III Nrg1 proteins containing the c-terminal intracellular domain. Therefore the maintenance of the two pools is most likely controlled by rates of proteolytic processing. We propose that *in vivo*, the former, back signaling-competent pool is preferentially found at nerve terminals where it is available for acute regulation of receptors such as TRPV1 and α7*nAChRs [2], [32].

We hypothesize that the endogenous activator of Type III Nrg1 back signaling at peripheral nerve terminals is either ErbB receptor expressed on keratinocytes or depolarization, as activity has been shown to activate Type III Nrg1 back signaling both *in vitro* [1] and *in vivo* [22]. The inability of Type III Nrg1^+/−^ cultures to acutely up-regulate levels of functional TRPV1 may explain why Type III Nrg1^+/−^ mice show profound reductions in their ability to develop capsaicin-induced inflammatory thermal hypersensitivity. Based on the strong association between the specificity of the pain phenotype seen in Type III Nrg1^+/−^ animals and the changes seen in sensory neurons from those animals, we conclude that the effect of Type III Nrg1 signaling on TRPV1 in sensory neurons is important for thermal pain sensation and the development of thermal hypersensitivity *in vivo*.

Our finding that Type III Nrg1 signaling in sensory neurons regulates functional levels of the receptor TRPV1 in a way that may affect thermal pain sensation and inflammatory thermal hypersensitivity also has implications for understanding the neurobiology of sensory abnormalities occurring in schizophrenia. Schizophrenia patients show abnormalities in pain sensitivity [12], [14], [16], [18], [35], but these deficits were often attributed to general cognitive deficits seen in the disease. We now provide evidence that Type III Nrg1—a protein whose levels were decreased in tissue from some schizophrenia patients—is important for the perception of thermal pain, and that this behavioral effect may result from regulation of functional axonal TRPV1 by Type III Nrg1 signaling in nociceptive sensory neurons. This finding, combined with prior results demonstrating regulation of axonal α7*nAChRs by Type III Nrg1 signaling [2], contributes to a growing body of work demonstrating that Type III Nrg1 is important for the function of nociceptive sensory neurons. In this way, we provide a means by which Type III Nrg1 signaling in the periphery could contribute to deficits in pain sensing seen in schizophrenia patients.
